# Short-term transplantation effect of a tissue-engineered meniscus constructed using drilled allogeneic acellular meniscus and BMSCs

**DOI:** 10.3389/fvets.2023.1266018

**Published:** 2023-11-17

**Authors:** Pengxiu Dai, Tong Zou, Wen Zhao, Yangou Lv, Dengke Gao, Chenmei Ruan, Xia Zhang, Xinke Zhang, Yihua Zhang

**Affiliations:** ^1^Shaanxi Branch of National Stem Cell Engineering and Technology Centre, College of Veterinary Medicine, Northwest A&F University, Xianyang, Shaanxi, China; ^2^Baiopai (Tianjin) Biotechnology Co., LTD, Tianjin, China

**Keywords:** drilled allogeneic acellular meniscus, porosity, BMSCs, drilled tissue-engineered meniscus, transplantation therapy

## Abstract

During the construction of tissue-engineered meniscus, the low porosity of extracellular matrix restricts the flow of nutrient solution and the migration and proliferation of cells, thus affecting the tissue remodeling after transplantation. In this study, the canine allogeneic meniscus was drilled first and then decellularized. The drilled tissue-engineered menisci (Drilled Allogeneic Acellular Meniscus + Bone Marrow Mesenchymal Stem Cells, BMSCs) were transplanted into the knee joints of model dogs. On the basis of ensuring the mechanical properties, the number of the porosity and the cells implanted in allogeneic acellular meniscus was significantly increased. The expression levels of glycosaminoglycans and type II collagen in the drilled tissue-engineered meniscus were also improved. It was determined that the animals in the experimental group recovered well-compared with those in the control group. The graft surface was covered with new cartilage, the retraction degree was small, and the tissue remodeling was good. The surface wear of the femoral condyle and tibial plateau cartilage was light. The results of this study showed that increasing the porosity of allogeneic meniscus by drilling could not only maintain the mechanical properties of the meniscus and increase the number of implanted cells but also promote cell proliferation and differentiation. After transplantation, the drilled tissue-engineered meniscus provided a good remodeling effect *in vivo* and played a positive role in repairing meniscal injury, protecting articular cartilage and restoring knee joint function.

## Introduction

The meniscus is a kind of C-shaped fibrous cartilage tissue. It is located between the femoral condyle and tibial plateau in the knee joint and has important physiological functions, such as protecting and stabilizing the knee joint, transmitting stress, absorbing impact, nourishing and lubricating the tissue, and preventing the degeneration of articular cartilage (1). Statistically, 60~70% of patients with knee injuries have meniscus tears, and the main clinical symptoms of meniscal injury are acute pain and dysfunction (2). At present, the treatment methods for meniscal injury are not sufficiently effective. Surgical suturing and repair are suitable for the treatment of meniscal injuries only in the peripheral vascular zone, and meniscal tearing recurs in one-third of cases after treatment (3). Meniscal injury in the avascular zone is treated by excision. Although meniscectomy provides temporary pain relief, it can lead to articular cartilage degeneration and cavity narrowing and accelerate the development of osteoarthritis by increasing the pressure (4). The transplantation of allografts or prostheses achieves partial effects. Nevertheless, allografts have not only the disadvantages of immune rejection and graft retraction but also a 29% failure rate (5), which is higher than that of cell-less meniscal stent transplantation (6). A comprehensive knowledge of the different approaches to meniscal substitution is required in order to integrate these evolving techniques in daily clinical practice to prevent the devastating effects of lost meniscal tissue (7, 8).

Recently, the rapid development of tissue engineering and regenerative medicine has brought new methods to the treatment of meniscal injury. DMECM (decellularized meniscal extracellular matrix) retains the bioactive substances and three-dimensional structure of the meniscus with certain characteristics, such as biomechanical properties to similar to those of the natural meniscus, good cell affinity and low immunogenicity (9, 10). Therefore, DMECM may be an ideal scaffold to construct tissue-engineered meniscal tissue. However, some studies found that the flow of nutrient solution and the migration and proliferation of cells were restricted due to the low porosity of the tissue-engineered meniscus constructed with DMECM, which affected tissue remodeling after transplantation into the body (11, 12). For this reason, the laboratory used formic acid, trypsin, and collagenase to treat allogeneic DMECM. Although the porosity and pore size of the treated allogeneic acellular meniscus increased, the structure of the collagen fibers in the meniscus was seriously damaged, and the biomechanical properties were significantly reduced. Therefore, these menisci were no longer suitable for incorporation into tissue-engineered meniscal tissue. The aim of this study is to investigate the feasibility of using a drilling method to increase the porosity of allogeneic DMECM and to determine the influence of drilling on the decellularization time of the meniscus. The study on the mechanical properties, the number of implanted cells and the repair effect of allogeneic acellular meniscus can provide reference for the application of tissue engineering meniscus.

## Methods

### Meniscus collection and drilling

**Figure 1:**
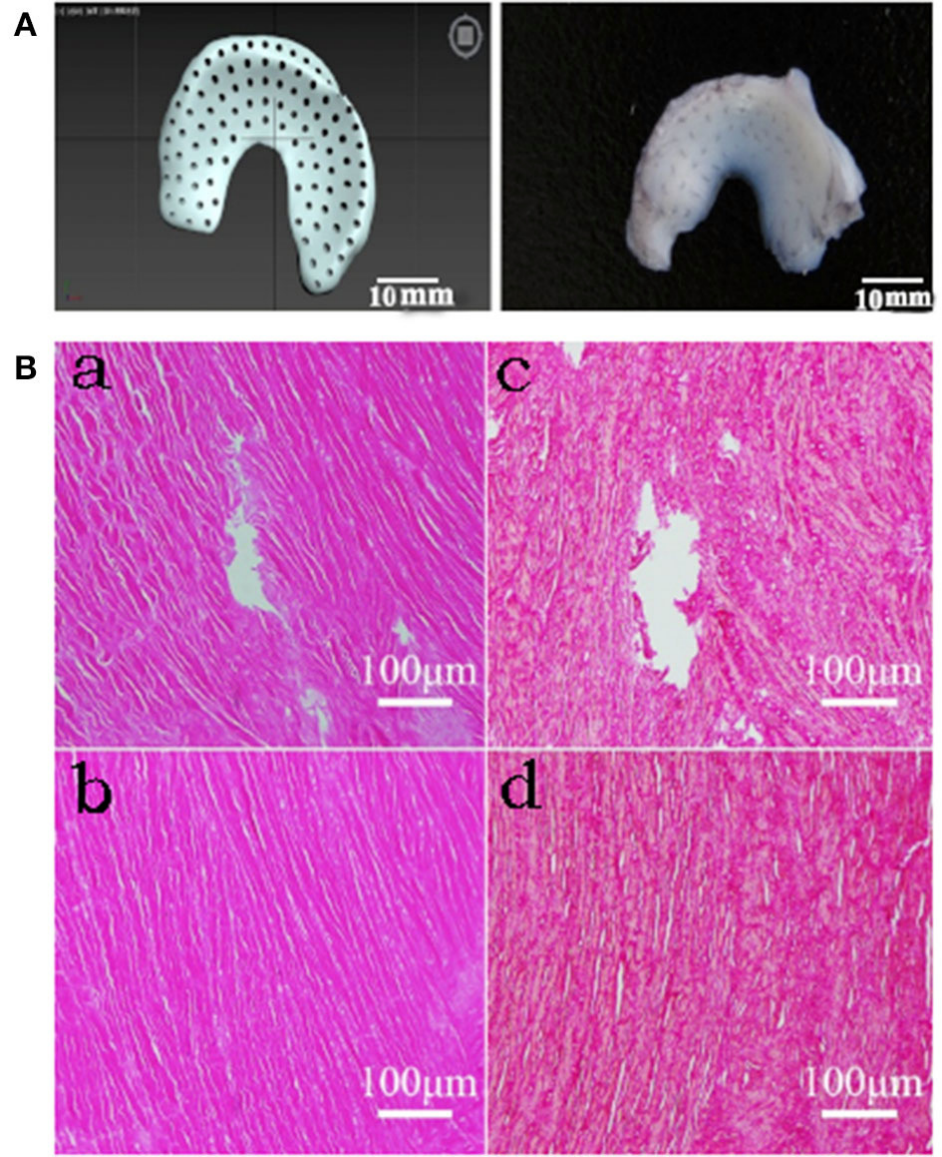
Histological results of drilled DMECM. **(A)** Schematic diagram of the drilled meniscus. **(B)** Histologic staining results of MECM scaffolds. (a, b) HE staining in drill group and control group respectively. (c, d) Sirius red staining in drill group and control group respectively. They show that cells have been completely removed in MECM and collagen fiber structure is complete around the hole.

A total of 120 medial menisci were collected from the knee joints of 60 deceased dogs provided by Experimental Animal Center of Northwest A&F University. Sixty of the menisci (30 from each side) were drilled perpendicular to the bottom of the meniscus with a medical multifunctional electric drill with a 26-g needle (diameter of 450 μm). According to the preliminary test results, the hole spacing was set to 1 mm (Figure 1A). The remaining 60 menisci were left as controls.

### Decellularization

According to the prescreening results from our laboratory, the menisci in the drill group and the control group, which underwent 3 freeze–thaw cycles, were shaken in a constant temperature shaking bed in decellularization fluid containing 1.0% (w/w) sodium dodecyl sulfate (SDS), 0.1% (w/v) ethylenediaminetetraacetic acid (EDTA) and 0.5% (w/w) peracetic acid. The decellularization fluid was changed every 2 days. According to the prescreening results from our laboratory, the undrilled group took longer to complete the decellularization due to the absence of voids. The drill group was treated for 3 days, and the control group was treated for 6 days. After removal of the decellularization fluid, the samples in both groups were washed with distilled water in an ultrasonic cleaner for 1 h, disinfected with 75% ethanol in a shaker for 1 h, washed with sterile Phosphate Buffer Saline (PBS) in a shaker for 1 day, wrapped with moist filter paper and stored at −20°C.

### DMECM histological evaluation

Allogeneic DMECM samples (*n* = 3) from the drill group and the control group were placed in 4% paraformaldehyde phosphate buffer solution at room temperature for 48 h. The samples were dehydrated by gradient alcohol, cleared by xylene and embedded in conventional paraffin wax. The sections were then continuously sliced at a thickness of 5 μM. The sections were stained with HE (hematoxylin and eosin) and Sirius scarlet and then observed under an optical microscope to determine whether the cells had been removed cleanly and the collagen fiber structure was intact.

### Allogeneic DMECM characterization

#### Porosity

The meniscus was placed in an electric drying oven to dry to constant weight. Under constant temperature conditions, the mass of the 5 mL specific gravity bottle filled with anhydrous ethanol was set as M_1_, and the dry reweight of the meniscus was set as M_0_. Allogeneic DMECM samples (*n* = 3) were obtained from the drill group and the control group, placed into the specific gravity bottles filled with anhydrous ethanol, allowed to stand for 24 h. Next, the samples were degassed with a vacuum pump until no bubbles escaped, and the bottles were filled with anhydrous ethanol and weighed as M_2_. The meniscus filled with anhydrous ethanol was removed, and the remaining anhydrous ethanol and the specific gravity bottle were weighed as M_3_. Porosity = (M_2_-M_3_-M_0_)/(M_1_-M_3_).

#### Compression modulus

Using a trephine, the meniscus was made into a cylinder with a diameter of 5.5 mm and a thickness of 2 mm. Two groups of allogeneic DMECM samples (*n* = 3) were placed on the loading platform of a Zwick 250 electronic universal testing machine (ZWICK, Germany). Each stent sample was preloaded with 1 N vertical pressure, and the compression test was carried out at a strain rate of 1 mm/min. The stress-strain curve is drawn with the deformation as the transverse axis and the pressure as the vertical axis. The compressive modulus of the meniscus was calculated according to the slope of the starting section of the curve and the cross-sectional area of the cylinder.

### Cytocompatibility of DMECM and BMSCs

The identified 4th passage canine BMSCs frozen in the laboratory (13, 14) were amplified for 2 passages using complete α-MEM (α-MEM culture medium containing 10% fetal bovine serum, 1% 100 × Penicillin Streptomycin Solution and 0.25% Anti-Myc Mycoplasma scavenging reagent) in 60 mm petri dishes. The 6th passage BMSCs were prepared into 3 × 10^5^/mL cell suspensions, and 500 μL of the cell suspensions were inoculated (uniform drop cloth) on the drilling meniscus (*n* = 3) and the control meniscus (*n* = 3). The cells were placed in the incubator for 5 h, and after sufficient adhesion to the meniscus, the cells were transferred to a 12-well plate for coculture with complete α-MEM at 37°C under 5% CO_2_ with saturated humidity. The medium was changed every 2 days.

#### Microstructure

The samples of the drilled meniscus (*n* = 3) and the control meniscus (*n* = 3) before and after implantation of the cells were placed in 2.5% glutaraldehyde at 4°C overnight, washed with PBS, dehydrated with an alcohol series, dried with CO_2_ critical point dryer for 4 h, sprayed with gold by an ion plating apparatus, and observed by scanning electron microscopy.

#### BMSC proliferation assay

The proliferation of BMSCs on the drilled meniscus and control meniscus was detected by the CCK-8 method. After 1, 4 and 7 days of coculture, the original complete α-MEM was replaced with new complete α-MEM containing 10% CCK-8. The cells were then placed in an incubator for an additional 4 h of culture. The OD_450_ value of the solution in each well was measured by a multifunctional enzyme marker (*n* = 3).

### Construction and induction of tissue-engineered meniscal tissue *in vitro*

Sixth passage canine BMSCs were prepared into a 3 × 10^6^/mL cell suspension, and 500 μL of the cell suspension was added dropwise to the upper surface of the drilled allogeneic DMECM. The meniscus was turned over after cell adhesion was fully achieved, and then 500 μL of cell suspension was evenly added to the underside of the meniscus, which was transferred to a 12-well plate after cell adhesion was fully achieved. After coculture at 37°C under 5% CO_2_ with saturated humidity, chondrogenic medium (α-MEM containing 10% FBS, 40 ng/mL dexamethasone, 50 μg/mL ascorbic acid, 50 μg/mL L-proline, 1 mmol/L sodium pyruvate, 10 μL/ml ITS, 10 ng/mL TGF-β3, 10 ng/mL BMP-2 and 1% double antibody) was used for culture on Day 3. The medium was changed every 2 days. The same treatment protocol was performed with undrilled allogeneic DMECM as controls.

### Detection of cell growth and differentiation degree in tissue-engineered meniscal tissue with different induction times

#### Glycosaminoglycan (GAG) determination

The drilled tissue-engineered meniscus (*n* = 3) from two groups induced for 1, 2, 3 and 4 weeks were digested overnight at 65°C with papain extract (8 mg/mL sodium acetate, 4 mg/mL disodium ethylenediamine tetraacetate, 0.8 mg/mL cysteine hydrochloride, 0.1 mg/mL papain). After centrifugation of the digestive solution for 10 min at 10,000 × g, the supernatant was removed. According to the instructions of the Blyscan™ Sulfated Glycosaminoglycan Assay kit (Biocolor, UK), the light absorption values were measured at 656 nm using a multifunctional enzyme marker. After subtracting the absorbance value of the empty allogeneic DMECM, the content of GAGs in samples from each group was calculated according to the standard curve.

#### Western blot analysis of type II collagen (COL-II)

The drilled tissue-engineered meniscus (*n* = 3) from the two groups induced *in vitro* for 1, 2, 3 and 4 weeks were cut up in an ice box. Total protein was extracted by RIPA, and the total protein concentration was determined by BCA. After the concentration of total protein in each group was adjusted, loading buffer was added to the samples for denaturation by boiling, SDS–PAGE electrophoresis, membrane transfer, sealing, and incubation with type II collagen antibody (Abcam, UK) and the corresponding secondary antibody. Finally, chemiluminescent solution was added to analyze the sample bands, and a gel image processing system was used to detect the relative expression level of COL-II in the samples.

### Drilled DMECM-BMSC tissue-engineered meniscal transplantation *in vivo*

Sixteen healthy dogs aged 2–5 years and weighing 6–8 kg (provided by the Laboratory Animal Center of Northwest A & F University) were randomly divided into the drill group, control group, defect group and sham operation group, with 4 dogs in each group. According to the ANOVA degrees of freedom (E) and the 3R principle, our E value is 12, which proves that the number of animals is sufficient. For Meniscus repair, we believe that four dogs can demonstrate the short-term transplantation effect of a tissue-engineered meniscus. All of the dogs were reared, obtained, and housed in accordance with our institute's laboratory animal requirements. All procedures and the study design were conducted in accordance with the Guide for the Care and Use of Laboratory Animals (Ministry of Science and Technology of China, 2006) and were approved by the Animal Ethical and Welfare Committee of Northwest Agriculture and Forest University.

The drill group underwent surgical resection of the medial meniscus of the right knee, and drilled tissue-engineered menisci were implanted after 4 weeks of induction *in vitro*. After general anesthesia [Zoletil^®^ 50 and Su-Mian-Xin II (1:1 mixture), 0.1 mL/kg intramuscular injection], the animals were placed in the supine position. Made a 10 cm skin incision along the tibial tuberosity. The subcutaneous fascia was bluntly separated. The joint capsule was cut, the medial collateral ligament was transected, and the medial meniscus was exposed and separated. Then the whole medial meniscus was resected, and the tibial platform graft bed was established. The tissue-engineered meniscus constructed *in vitro* was placed into the graft bed. The anterior corner of the meniscus was fixed between the tibial condyles with 4-0 PGA line, the posterior corner was fixed in front of the medial intercondylar spine and posterior cruciate ligament insertion, and the body was fixed in the articular capsule. The joint capsule, fascia and skin were routinely closed after suturing the ligaments. After the operation was completed, the surgical site was disinfected. The surgical part was isolated with sterile gauze, and the limbs were wrapped with medical cotton and immobilized by polymer splints. In the control group, the medial meniscus of the right knee was surgically removed and implanted with the undrilled DMECM-BMSC tissue-engineered meniscus after 4 weeks of induction *in vitro*. In the defect group, the medial meniscus of the right knee was surgically resected, and the incision was sutured directly without repair. In the sham operation group, the joint capsule of the right knee joint was incised, and the surgical incision was sutured directly without cutting the meniscus. After surgery, the right knee joint was wrapped with medical cotton and immobilized with a splint. Synulox RTU (Zoetis, USA) was injected intramuscularly for 3 consecutive days to prevent infection, and carprofen (Pfizer Animal Health B.V., USA) was injected subcutaneously to control postoperative pain. The splint was removed 7 days later, and the skin sutures were removed 10 days later. The kennel was ventilated and disinfected regularly, and the test dogs exercised regularly.

### Detection and evaluation of the repair effect of the drilled DMECM-BMSC tissue-engineered meniscus after *in vivo* transplantation

#### Postoperative observations and knee function score

The recovery of motor function of the test dogs in each group was observed and recorded continuously after surgery. At 2 months after surgery, three experienced clinical veterinarians who were unaware of the experimental groupings or treatments were invited to perform Lysholm knee scoring for the test dogs in each group (*n* = 4) based on eight indices, including lameness, support, locking, instability, pain, swelling, stair climbing and squatting (15).

#### Knee dissection and histological score

The dogs in each group were euthanized 12 weeks after surgery, the articular cavity was opened according to the transplantation path, and changes in the meniscal graft, femoral condyle and tibial plateau cartilage were observed. A Vernier caliper was used to measure the width at 1/2 of the center of the meniscal graft (W_1_), and the width of the tissue-engineered meniscus before transplantation was recorded as W_0_. Then, the width retraction rate of the transplanted meniscus was calculated as follows: (W_0_-W_1_)/W_0_. Three experienced pathology technicians who were not aware of the group conditions or repair measures were asked to report which symptoms were observed according to the gross evaluation criteria of the International Cartilage Repair Society (ICRS) (16). Gross observation scores (0–24 points) were calculated on the knee joints of the test dogs from four aspects: the degree of defect repair, the degree of integration between the graft and peripheral tissues, the gross appearance of the surface, and evaluation of the overall repair. The higher the total score was, the closer the graft was to the normal meniscus.

#### Histological observation of the knee joint

Meniscal graft samples were cut from the knee joint and fixed with 4% paraformaldehyde for 48 h. Then, paraffin sections were made, and hematoxylin-eosin (HE) staining was performed to observe the tissue structure. Toluidine blue (TB) staining was performed to observe the acid polysaccharide in the matrix. Chondroproteoglycan (ACAN) and COL-II antibodies were used for immunohistochemical staining to observe the expression and distribution of ACAN and COL-II.

Samples of the femoral condyle, tibial platform cartilage and subchondral bone were cut from the knee joint and placed in EDTA-2Na solution for decalcification. The decalcification solution was replaced every 3 days. After complete decalcification, paraffin sections were made, and HE and Safranin O-Fast green (SO-FG) staining were performed to observe the changes in cartilage.

### Statistical analysis

The experimental data were processed by SPSS 18.0 software, and the results are expressed as the mean ± standard deviation. Comparisons between groups were conducted by One-Way ANOVA. *P* < 0.05 was considered a significant difference, and *P* < 0.01 was considered an extremely significant difference. Bar graphs were constructed using GraphPad Prism 6 software.

## Results

### Histological assessments of the drilled DMECM

HE-stained sections were observed by optical microscopy. No nuclei were found in the allogeneic DMECM in the drilled group or control group, indicating that the cells had been cleanly removed. The Sirius scarlet staining results showed that the collagen fiber structure in the allogeneic DMECM was complete. An obvious hole-like structure was observed in the meniscus in the drill group, and the collagen fiber structure around the pore wall was complete (Figure 1B).

### Porosity and compression modulus of the drilled DMECM

The results of the porosity and compression modulus assessments of the drilled and control meniscus are shown in Table 1. The porosity of the drilled DMECM (56.93 ± 0.86) was significantly higher than that of the control group (31.58 ± 1.45) (*P* < 0.01). The compression moduli of the drilled DMECM (51.63 ± 0.89) and control DMECM (52.51 ± 1.24) were not significantly different from that of the normal meniscus (54.14 ± 1.39) (*P* > 0.05), indicating that drilling had no significant effect on the mechanical properties of the meniscus.

**Table 1:** Test results of porosity and compressive modulus of the scaffolds.

	**Drill group**	**Control group**	**Natural meniscus**
Porosity/%	56.93 ± 0.86^**^	31.58 ± 1.45	—
Compressive modulus/Mpa	51.63 ± 0.89	52.51 ± 1.24	54.14 ± 1.39

x ± s, n = 3. The porosity of drilled DMECM scaffold is significantly higher than the control group, ^**^P < 0.01.

### Cell affinity of the drilled DMECM

**Figure 2:**
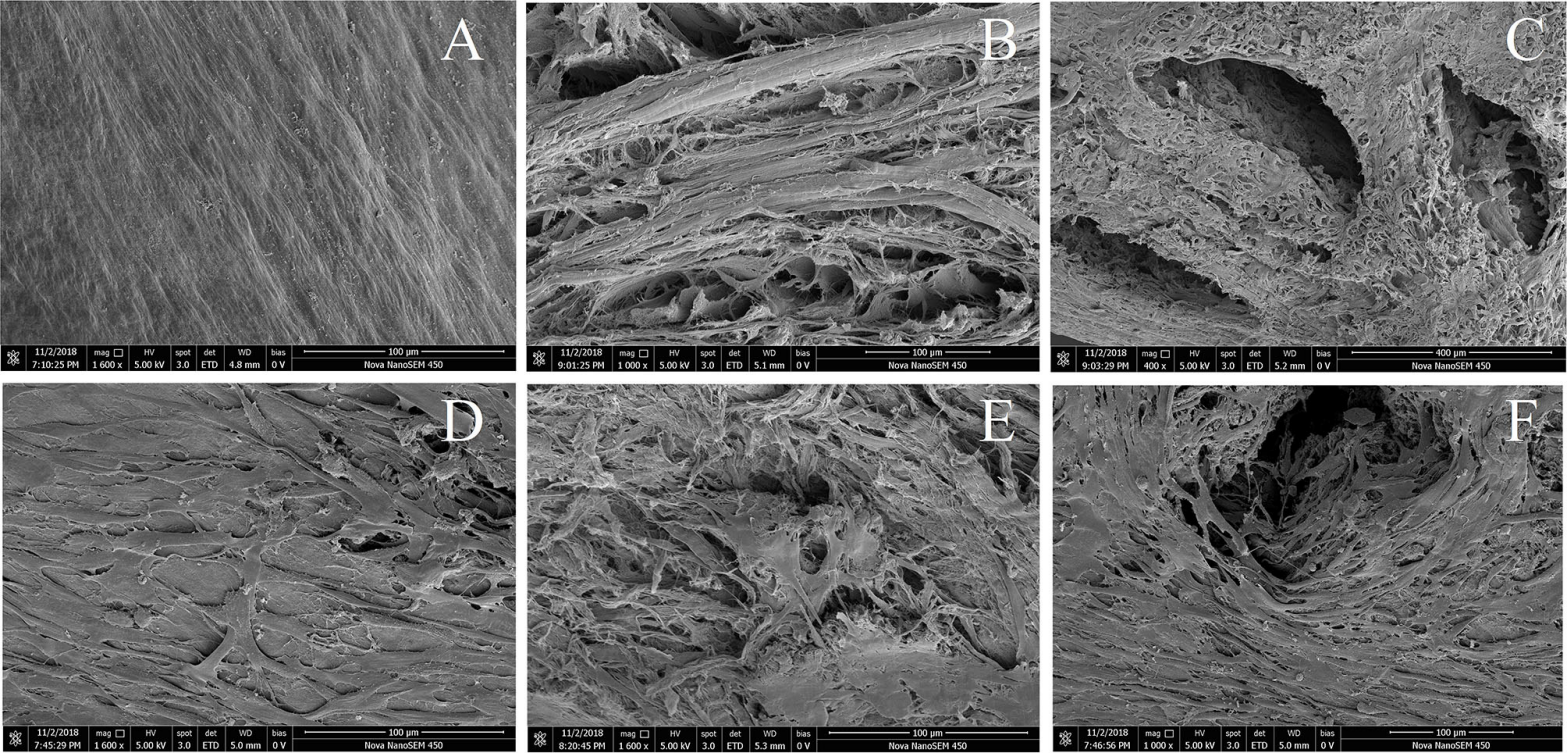
Scanning electron microscope observation of DMECM before and after implantation of BMSCs. **(A)** Dense surface of DMECM, no obvious pores. **(B)** Loose subface of DMECM with many small pores. **(C)** Subface of drilled DMECM, the matrix surrounding the holes is structurally intact. **(D)** Growth of BMSCs on the surface of DMECM. **(E)** Growth of BMSCs on the subface and inside the pores of DMECM. **(F)** Growth of BMSCs into the hole of drilled DMECM.

As observed by scanning electron microscopy, the surface structure of the control DMECM was dense with no obvious pores (Figure 2A), while the microstructure of the bottom surface was relatively loose with many small pores (Figure 2B). The drilled DMECM had holes with diameters of more than 400 microns, and the matrix surrounding the holes was structurally intact (Figure 2C). After BMSCs were implanted into the meniscus, the surface (Figure 2D) and the bottom of the control DMECM was covered with cells, and some cells grew into the pores (Figure 2E). The holes of the drilled DMECM were overgrown with cells, and a large number of cells grew into the holes (Figure 2F), while cell growth in the other parts of the meniscus was similar to that in the control group.

The results of BMSC proliferation in the DMECM are shown in Table 2. The OD_450_ value of the BMSC-DMECM complex in the drill group and control group after 1, 4 and 7 days of culture showed an increasing trend with time, but the OD value in the drill group was significantly higher than that in the control group after 1 day of culture (*P* < 0.05) and was extremely significantly higher than that in the control group after 4 and 7 days of culture (*P* < 0.01). These results indicated that drilling could not only increase the number of cells planted in the meniscus but also facilitate the growth and reproduction of the implanted cells.

**Table 2:** Results of CCK-8 proliferation test.

**Group/time (day)**	**1**	**4**	**7**
Drill group	0.525 ± 0.028^*^	0.774 ± 0.025^**^	0.980 ± 0.029^**^
Control group	0.405 ± 0.023	0.497 ± 0.019	0.538 ± 0.021

x ± s, n = 3. OD value in drill group was significantly higher than that in control group at 1 day of culture, ^*^P < 0.05. OD value in drill group was extremely significantly higher than that in control group at 4 and 7 days of culture, ^**^P < 0.01.

### Cell differentiation in the tissue-engineered meniscus after different induction times

The GAG contents (μg/mg, wet weight) in the drilled and control tissue-engineered meniscus after 1, 2, 3, and 4 weeks of induction *in vitro* are shown in Table 3. The GAG contents in both groups showed an increasing trend with increasing induction time, but the GAG content in the drill group was significantly higher than that in the control group after 2 weeks of induction (*P* < 0.05) and was extremely significantly higher than that in the control group after 3 and 4 weeks of induction (*P* < 0.01).

**Table 3:** GAG quantitative detection result of cell-scaffold complexes.

**Group/time (week)**	**1**	**2**	**3**	**4**
Drill group	0.921 ± 0.068	2.146 ± 0.126^*^	3.725 ± 0.125^**^	4.606 ± 0.115^**^
Control group	0.847 ± 0.110	1.487 ± 0.116	2.216 ± 0.208	2.710 ± 0.107

x ± s, n = 3. The GAG content in drill group was significantly higher than that in control group at 2 weeks of induction, ^*^P < 0.05. The GAG content in drill group was extremely significantly higher than that in control group at 3 and 4 weeks of induction, ^**^P < 0.01.

**Figure 3:**
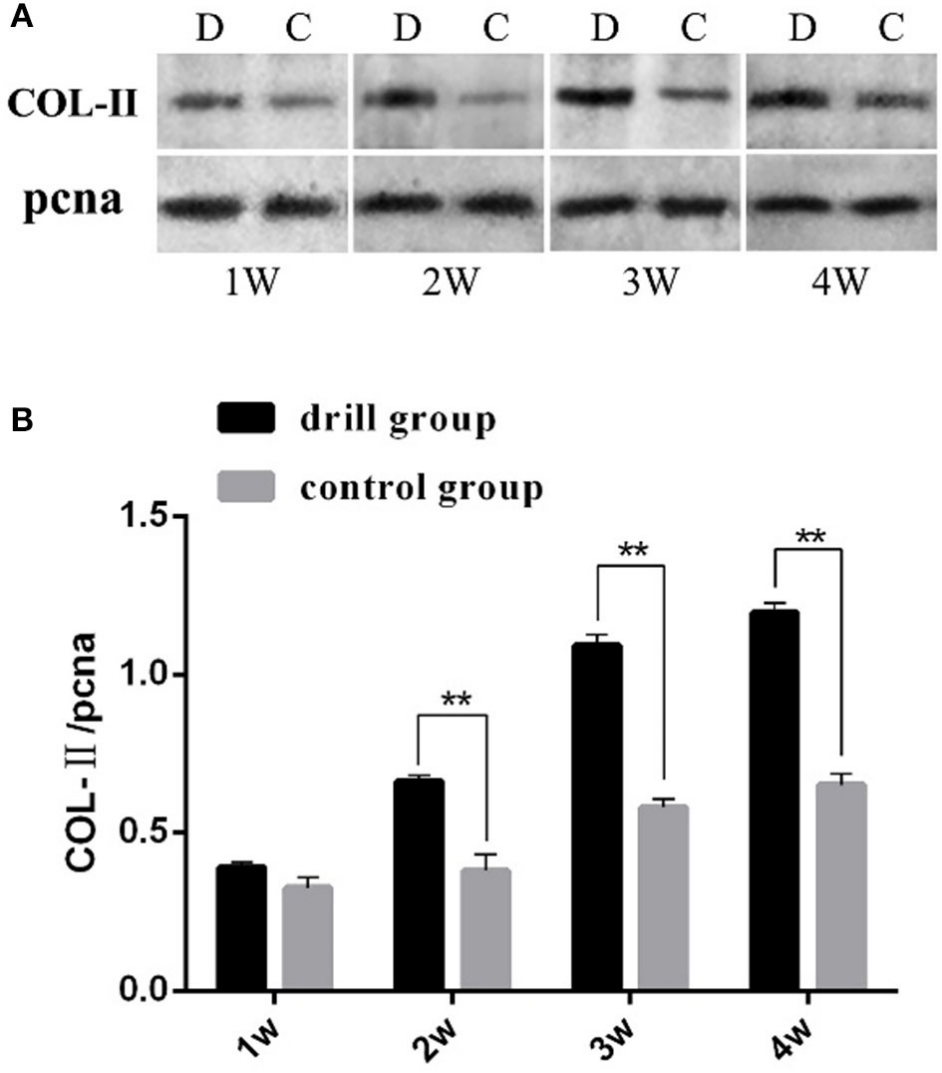
Western blot analysis of type II collagen in engineeringed meniscus. **(A)** Gel electrophoresis results, “pcna” is the internal reference, D represents drill group, C represents control group. **(B)** Analysis results of band gray scale, “**” mean *P* < 0.01.

The COL-II Western blot results showed that both the drill group and the control group expressed COL-II, and the gray value showed an increasing trend with increasing induction time. However, the relative expression level in the drill group was extremely significantly higher than that in the control group after 2, 3 and 4 weeks of induction (*P* < 0.01) (Figure 3). These results indicate that drilling is beneficial for cell differentiation in the cartilage within the meniscus.

### Detection and evaluation of the repair effect of the drilled tissue-engineered meniscus *in vivo*

#### Postoperative observation and knee function score results

On the second day after surgery, the mental state, appetite and other signs of the dogs in each group returned to normal, although some had slight swelling of the operated limb. After 10 days, the incisions had healed well, except for those in the sham operation group, and the other 3 groups still displayed slight swelling of the knee joint. Two weeks after the operation, the dogs in the drill group and the control group tended to stand, squat, walk and step up and down normally, with no obvious claudication or interlocking phenomena; knee swelling disappeared, and only slight claudication occurred during vigorous running.

After 4 weeks, all motor functions returned to normal. The test dogs in the defect group showed lameness at all times after surgery, weight loss in the operated limbs, stiff movement of the knee joint, difficulty climbing up and down steps, inability to jump. The dogs in the sham operation group returned to normal standing, squatting, walking and stepping 2 weeks after surgery, and no abnormalities were observed in the operated limbs or knee joints. Table 4 shows the Lysholm score of knee function in each group 2 months after surgery. The score in the drill group (86.15 ± 1.56) was higher than that of the control group (81.36 ± 0.98) (*P* < 0.01) and was closest to that of the sham group (97.19 ± 0.95) (*P* < 0.01). The scores of the drill group and the control group were significantly higher than those of the defect group (24.26 ± 1.77) (*P* < 0.01).

**Table 4:** Lysholm score results.

**Group**	**A dog**	**B dog**	**C dog**	**D dog**	**‘x ±s**
Drilling	84.66	87.34	84.46	88.12	86.15 ± 1.86^##^
Control	80.46	82.58	81.72	80.67	81.36 ± 0.98^**^
Model	24.60	23.36	26.58	22.48	24.26 ± 1.77
Sham	96.90	97.83	98.04	95.98	97.19 ± 0.95^∧∧^

The score of the drill group was higher than that of the control group, ^##^P < 0.01, and was closest to that of the sham group, ^∧∧^P < 0.01. The score of the control group was significantly higher than those of the defect group, ^**^P < 0.01.

#### Knee dissection and ICRS score

**Figure 4:**
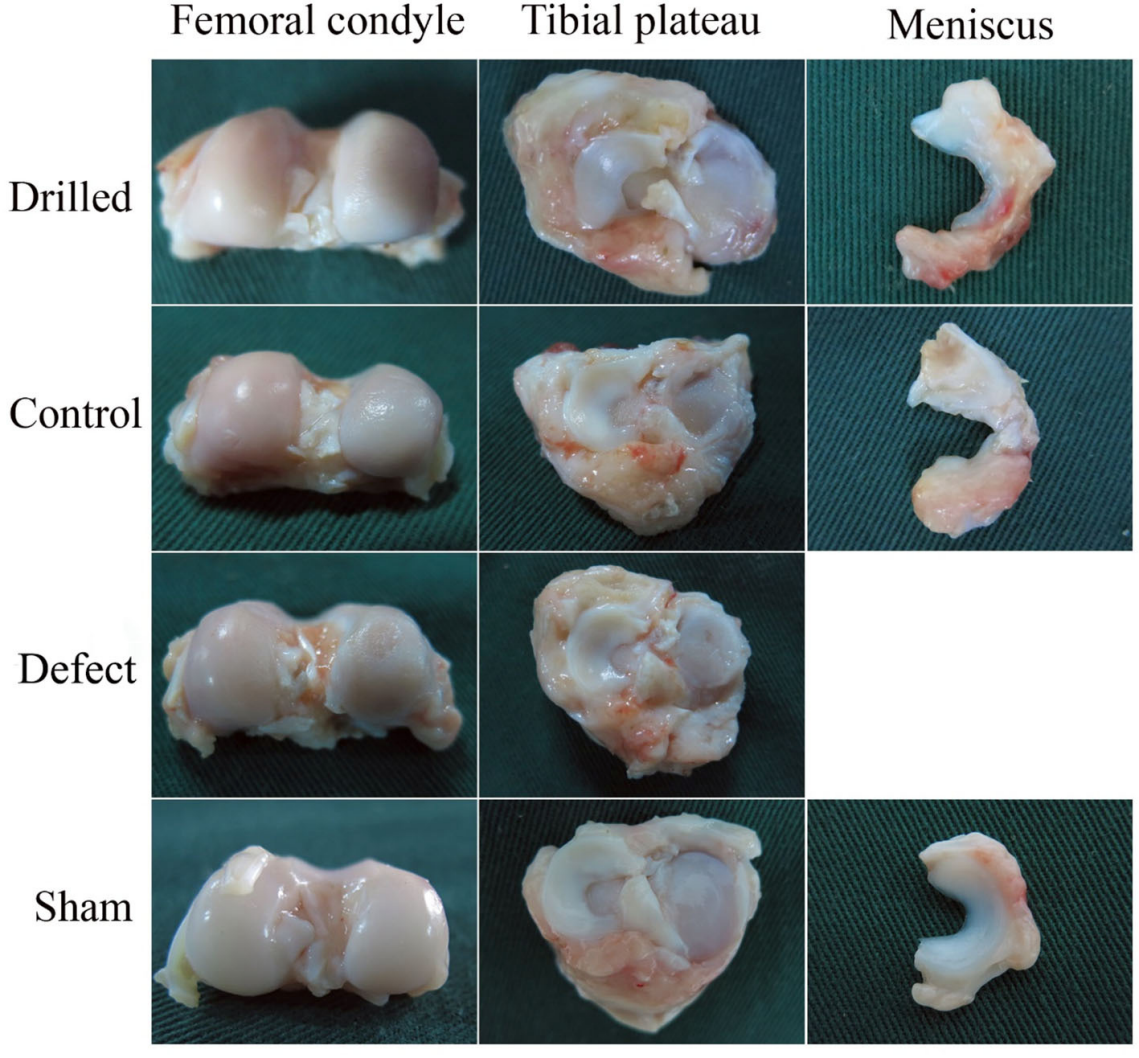
Anatomical observation of the knee joint. 12 weeks after surgery, the anatomical observation results of meniscus graft and knee cartilage of experimental dogs in each group.

The anatomical observation results of the meniscal grafts and knee cartilage of the experimental dogs in each group 12 weeks after surgery are shown in Figure 4. No obvious abnormalities were observed in the articular cavity of the drill group, and the joint fluid was clear and yellowish in color. The meniscal graft was complete, the outer edge and fore and aft angles of the graft were wrapped by the proliferative tissue of the joint capsule, the color was yellowish, and the internal surface consisted of translucent neoflochondroid tissue. The femoral condylar cartilage and tibial plateau cartilage were only slightly worn, and the surface was slightly rough and slightly darkened.

In the control group, the joint fluid was clear and yellow in color. The meniscal graft was completely wrapped by hyperplastic tissue, and the graft had an obvious retraction phenomenon. The degree of wear of the femoral condyle and tibial plateau cartilage was more serious than that of the drill group. In the defect group, the joint capsule was hyperplasic and thickened, the joint fluid was thick and yellow, and there were obvious wear and scratches on the surface of the femoral condylar cartilage and the tibial plateau cartilage. In the sham operation group, no obvious abnormalities were observed in the articular cavity, the joint fluid was clear and yellowish in color, no significant changes were observed in the meniscus or surrounding tissues, and the surfaces of the femoral condylar cartilage and tibial plateau cartilage were smooth and complete.

The results of the width retraction of the meniscal grafts are shown in Table 5. The retraction rate of the meniscal graft width in the drill group (14.79 ± 1.43%) was significantly lower than that in the control group (32.03 ± 1.22%) (*P* < 0.01). Meniscus width did not change in the sham operation group, and the retraction rate was 0.

**Table 5:** The width shrinkage ratio of meniscus graft (%).

**Group**	**A dog**	**B dog**	**C dog**	**D dog**	**‘x ±s**
Drilling (%)	16.78	13.86	14.88	13.65	14.79 ± 1.43
Control (%)	33.46	32.57	31.35	30.73	32.03 ± 1.22^**^
Sham (%)	0	0	0	0	0

The retraction rate of meniscus graft width in the drill group was significantly lower than that in the control group, ^**^P < 0.01. Meniscus width did not change in the sham operation group, and the retraction meter was 0.

The ICRS score results of the knee cartilage are shown in Table 6. The degree of articular cartilage injury in the drill group (0.31 ± 0.50) was significantly lower than that in the control group (1.04 ± 0.32) (*P* < 0.01), close to that in the sham group (0.21 ± 0.32), and significantly lower than that in the defect group (2.49 ± 0.65) (*P* < 0.01).

**Table 6:** ICRS score results.

**Group**	**A dog**	**B dog**	**C dog**	**D dog**	**‘x ±s**
Drilling	0.32	0.30	0.33	0.29	0.31 ± 0.50
Control	1.01	1.04	1.07	1.00	1.04 ± 0.32^##^
Model	2.39	2.53	2.33	2.65	2.49 ± 0.65^**^
Sham	0.19	0.25	0.22	0.18	0.21 ± 0.32

The injury degree of articular cartilage in the drill group was significantly lower than that in the control group, ^##^P < 0.01, close to the sham group, and significantly lower than the defect group, ^**^P < 0.01.

#### Histological observation of the knee joint

**Figure 5:**
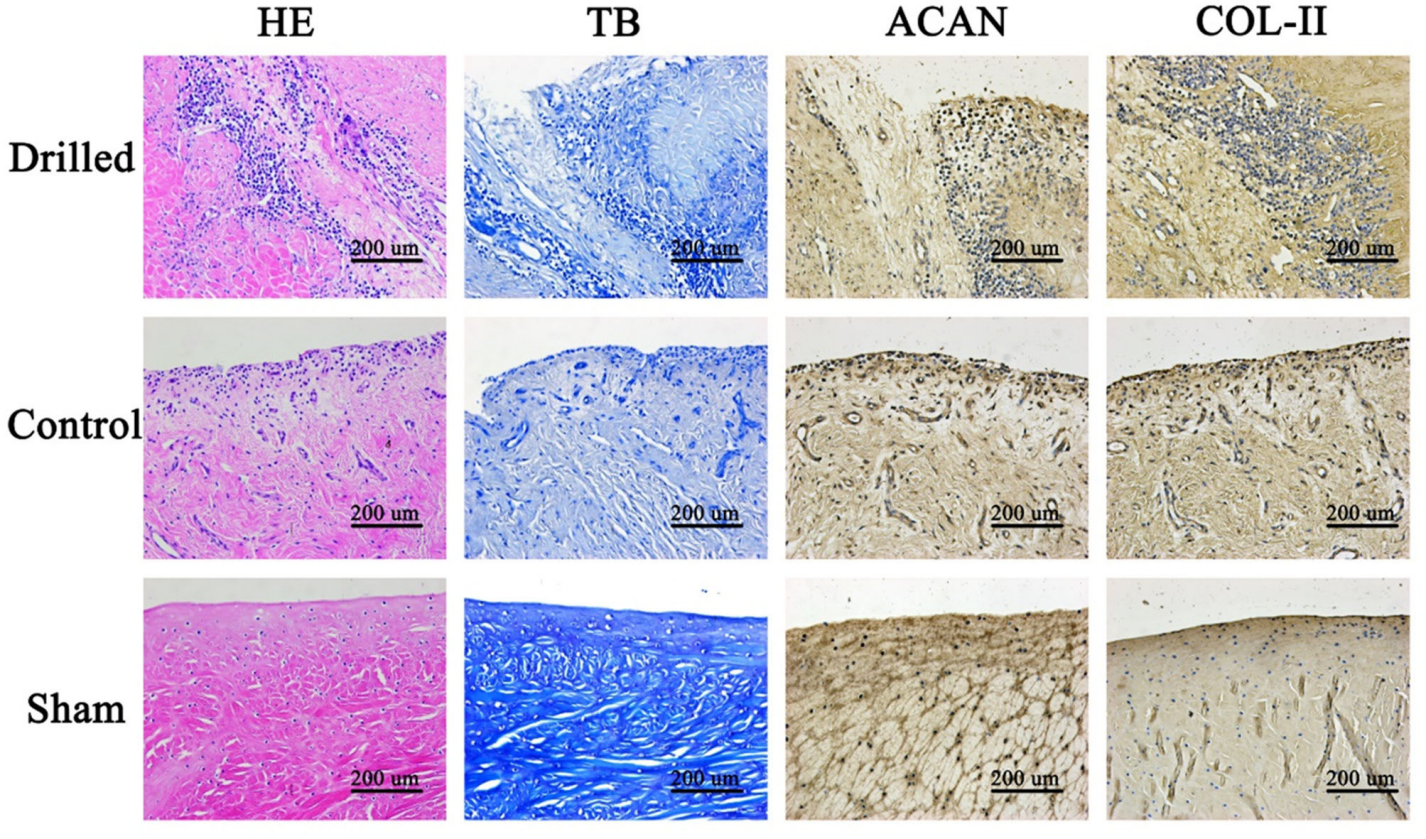
Histological observation of meniscus graft. In the drill group, a large number of cells grew along the channels and infiltrated into the surrounding matrix, generally migrating from the surface to the deep layer. The cell matrix around the pores in the drill group was darker, with a large number of blue-stained acidic polysaccharides in the matrix, while that in the control group was lighter. In the drill group, there were more cells growing along the meniscus channel, more ACAN and COL-II around the cells, and the staining was darker. HE, Hematoxylin-eosin staining; TB, Toluidine blue staining; ACAN, Immunohistochemical staining for ACAN; COL-II, Immunohistochemical staining for COL-II. Drilled, the drill group; Control, the control group; Sham, the sham operation group.

Under the microscope, the HE-stained sections of the meniscal grafts in the drilled group showed obvious channels extending from the surface into the interior, and a large number of cells grew along the channels and infiltrated into the surrounding matrix, generally migrating from the surface to the deep layer. In the control group, the HE-stained sections of the meniscal grafts showed cell growth mostly on the surface or superficial layer, and the internal cells were few and scattered. At high magnification, monocytes infiltrated the surface of the implant, new vascular structures were observed in the stroma, and a small number of fibroid and chondroid cells were seen. TB staining showed that the cell matrix around the pores in the drill group was darker with a large number of blue-stained acidic polysaccharides in the matrix, while the cell matrix in the control group was lighter (Figure 5).

Immunohistochemical staining of meniscal grafts: ACAN and COL-II were stained brown in the matrix and distributed around the cells in a cell-dependent manner. In the drilled group, there were more cells growing along the meniscus channel and more ACAN and COL-II around the cells, appearing as darker staining. The control group had darker staining on only the surface or superficial layer (Figure 5).

**Figure 6:**
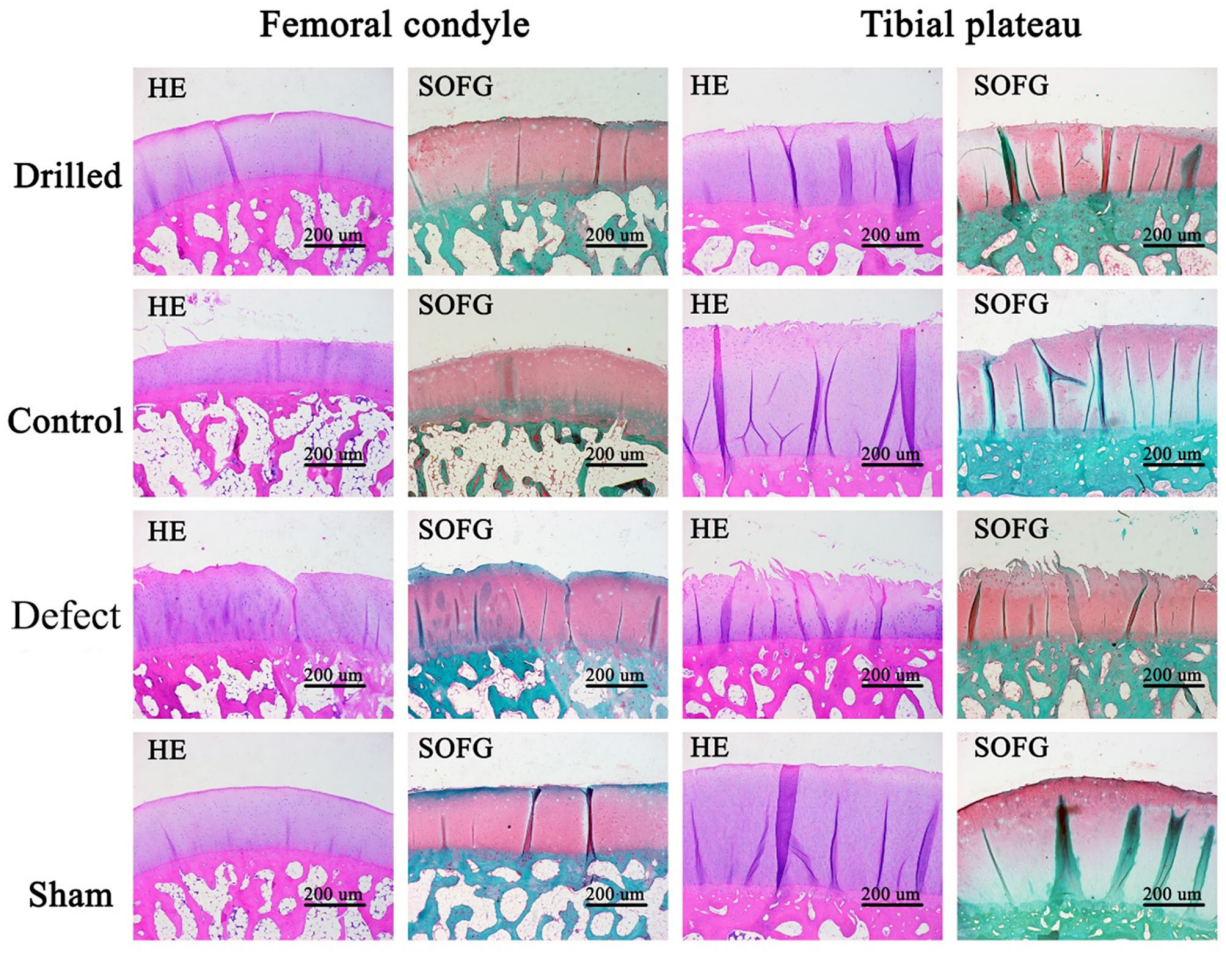
HE and SO-FG staining results of femoral condyle and tibial plateau. Compared with the control group, the surface of the drill group was basically smooth and flat, with slight wear, complete and clear tidal line, uniform matrix staining, and more ovate chondrocytes, which was closer to the sham operation group. In the defect group, the surface of the cartilage was severely damaged, rough and irregular cracks, and the chondrocytes and matrix were significantly reduced. Drilled, the drill group; Control, the control group; Defect, the defect group; Sham, the sham operation group. Femoral condyle, HE and SO-FG staining of femoral condyle; Tibial plateau, HE and SO-FG staining of tibial plateau.

Histological analysis of the femoral condyle and tibial plateau cartilage: HE- and SO-FG-stained sections of decalcified femoral condyle and tibial plateau cartilage samples were observed under a microscope. Compared with the control group, the surface of the drilled group was basically smooth and flat with slight wear, a complete and clear tidal line, uniform matrix staining, and more ovate chondrocytes, making it more similar to the sham operation group. In the defect group, the surface of the cartilage was severely damaged, rough and irregular cracks were observed, and the number of chondrocytes and matrix was significantly reduced (Figure 6).

## Discussion

The porosity and number of attached cells are important indices for the evaluation of scaffolds used in tissue engineering. Higher porosity of a scaffold is conducive to the migration and proliferation of cells into the deep layer of the scaffold, as it promotes the transport of nutrients and metabolites and communication between cells. The meniscus contains fewer cells and has a high density of collagen fibers in its matrix, so its porosity is low. Low porosity of a scaffold has a direct effect on the number of seed cells implanted and the metabolism and proliferation of seed cells for tissue remodeling. To this end, researchers have adopted a variety of methods to improve the porosity of DMECM scaffolds. Maier used a trypsin/collagenase/protease multistep digestion process to remove cells in the menisci of sheep, and the results showed that the porosity of the prepared DMECM scaffold increased (17). Chen et al. treated pig meniscal tissue with formic acid, acetic acid, peracetic acid, malic acid, succinic acid, citric acid and other acids, and the results showed that a higher concentration of acid solution and a shorter treatment time could improve scaffold porosity and achieve the best decellularization effect (18). In addition, some researchers have used laser ablation technology to change the porosity of DMECM scaffolds, but laser ablation can affect the structure of the peripore wall and prevent the diffusion of cells from the pore into the peripore wall. In this study, a drilling method was used to increase the porosity of the meniscal scaffold, which is a simple and convenient method that did not affect the peripore wall structure. It not only shortened the decellularization time of the meniscus but also improved BMSC deposition, adhesion, proliferation and chondroid differentiation in the scaffold (19).

The meniscus plays an important mechanical role in in the knee joint by transferring loads, reducing shock and absorbing stress (12). Therefore, scaffolds used to construct tissue-engineered meniscal tissue must have sufficient mechanical strength. DMECM stents are derived from animal knee meniscal tissue and have inherent mechanical properties. Notably, the mechanical properties of scaffolds are directly related to their porosity. To improve the cell implantation rate into DMECM scaffolds, artificially increasing the porosity will inevitably affect the mechanical properties and cell recombination ability of the scaffolds.

The meniscus plays an important role in the overall function of the knee joint. When meniscal damage is beyond repair, meniscal transplantation is an excellent alternative solution. Transplantation has been shown to reduce pain and improve knee function, as well as prevent cartilage degeneration and the development of osteoarthritis. Compared with the lateral meniscus of the knee, the medial meniscus is more prone to tear because it is tightly anchored to the tibial plateau by the coronary ligament and has a smaller range of motion. Therefore, in this study, a model of medial meniscus defect in the knee joint was first established, and then the tissue-engineered meniscus constructed *in vitro* was implanted into the recipient knee joint to test its repair effect. Meniscus size is specific to the particular individual. A donor graft that is too large can put more pressure on the joint surface, while one that is too small can put more pressure on itself (20). In this experiment, after collecting menisci from donor dogs in an early stage, determination of the relationship between meniscus size and donor dog body weight, leg circumference at the knee joint, tibial plateau size and other factors was attempted to provide selection criteria for the later recipient dogs and ensure matching of the donor and recipient dogs. However, there are still difficulties in terms of the practical operation, and further studies are needed to narrow the size gap between the donor and recipient as much as possible and maintain it within a controllable range to avoid transplantation problems caused by excessive size mismatch.

With the anatomical structure of the meniscus having a concave upper surface and a flat bottom and the histological structure of high-density collagen fibers, the meniscus converts the vertical pressure from the convex femoral condyle into horizontal circumferential stress that is evenly distributed throughout the tibial plateau, which increases the joint contact area and reduces the direct pressure on the joint surface, ultimately protecting the articular cartilage well (18). As the function of the meniscus was not previously recognized, meniscectomy or partial meniscectomy was usually performed for meniscal injury (21). Although these procedures alleviate symptoms in the short term, they seriously damage the articular cartilage in the long term and accelerate the development of osteoarthritis (22). Therefore, for meniscal injury, meniscal function should be retained and restored in principle. Because meniscal injuries are difficult to cure, patients can only seek replacement to maintain knee joint function. This involves meniscal transplantation. Currently, there is much research on meniscus repair. The methods of meniscal prosthesis transplantation, meniscal allotransplantation and tissue-engineered meniscal transplantation, which has developed rapidly in recent years, have obtained different degrees of therapeutic efficacy on meniscal injury, showing good protective effects on articular cartilage (23, 24). In this study, dogs underwent meniscectomy, and wear of the femoral condylar cartilage and tibial plateau cartilage were clearly observed in the knee anatomy 12 weeks after surgery; moreover, the surface was rough and dull. Histologically, the cartilage surface was discontinuous with varying degrees of fissures. In the defect model dogs implanted with the tissue-engineered meniscus, although slight wear of the femoral condyle and tibial plateau cartilage was observed anatomically, it was significantly better than that in the excised group. Histological assessments also showed that the cartilage surface maintained some integrity, indicating the protective effect of the meniscal graft on articular cartilage. However, the transplantation in this study was only 12 weeks, which has some limitations (cell growth/collagen production) for the observation of long-term transplantation effect. In the subsequent experiment, we will extend the transplantation time to explore the long-term transplantation effect.

In conclusion, the cell implantation rate of DMECM is a difficulty that remains in the development and utilization of tissue engineering. In this study, physical drilling of the meniscus was performed to improve the porosity of the DMECM on the premise of maintaining adequate mechanical properties, which not only shortened the decellularization time when preparing the scaffolds but also improved the implantation rate of the cells in scaffolds and the fluidity of culture medium. These factors were conducive to the deposition, adhesion, proliferation and differentiation of cells in scaffolds, thus promoting tissue remodeling *in vivo*. This strategy has positive significance in the recovery of knee joint function and lays a foundation for the extensive application of DMECM stents in the construction of tissue-engineered menisci. At the same time, the results of this study also provide a reference for human clinical tissue engineering meniscus and meniscus repair.

## Conclusions

Drilling can not only increase the porosity of DMECM, maintain the mechanical properties of these DMECM and increase the number of implanted cells but also promote the proliferation and differentiation of the cells and allow tissue remodeling after transplantation. The drilled tissue-engineered meniscus graft presented here has a positive effect on repairing meniscus injury, protecting articular cartilage and restoring knee joint function.

## Data availability statement

The original contributions presented in the study are included in the article/supplementary material, further inquiries can be directed to the corresponding author.

## Ethics statement

The animal study was approved by Animal Ethical and Welfare Committee of Northwest Agriculture and Forest University. The study was conducted in accordance with the local legislation and institutional requirements.

## Author contributions

PD: Data curation, Methodology, Supervision, Writing—original draft, Writing—review & editing. TZ: Data curation, Methodology, Validation, Writing—original draft. WZ: Data curation, Methodology, Validation, Writing—original draft. YL: Data curation, Validation, Writing—original draft. DG: Data curation, Validation, Writing—original draft. CR: Data curation, Validation, Writing—original draft. XiaZ: Data curation, Validation, Writing—original draft. XinZ: Data curation, Validation, Writing—original draft. YZ: Funding acquisition, Methodology, Project administration, Writing—original draft, Writing—review & editing.
